# Risk‐based versus universal PrEP delivery during pregnancy: a cluster randomized trial in Western Kenya from 2018 to 2019

**DOI:** 10.1002/jia2.26061

**Published:** 2023-02-20

**Authors:** John Kinuthia, Julia C. Dettinger, Joshua Stern, Nancy Ngumbau, Ben Ochieng, Laurén Gómez, Felix Abuna, Salphine Watoyi, Mary Marwa, Daniel Odinga, Anjuli D. Wagner, Barbra A. Richardson, Jillian Pintye, Jared M. Baeten, Grace John‐Stewart

**Affiliations:** ^1^ Department of Research and Programs Kenyatta National Hospital Nairobi Kenya; ^2^ Department of Global Health University of Washington Seattle Washington USA; ^3^ Department of Biostatistics University of Washington Seattle Washington USA; ^4^ Division of Allergy and Infectious Diseases University of Washington Seattle Washington USA; ^5^ Vaccine and Infectious Disease Division Fred Hutchinson Cancer Research Center Seattle Washington USA; ^6^ Department of Behavioral Nursing & Health Informatics University of Washington Seattle Washington USA; ^7^ Department of Epidemiology University of Washington Seattle Washington USA; ^8^ Department of Medicine University of Washington Seattle Washington USA; ^9^ Gilead Sciences Foster City California USA; ^10^ Department of Pediatrics University of Washington Seattle Washington USA

**Keywords:** pre‐exposure prophylaxis, pregnancy, postpartum, HIV prevention, breastfeeding, Kenya

## Abstract

**Introduction:**

Integrating pre‐exposure prophylaxis (PrEP) delivery for pregnant and postpartum women within maternal and child health (MCH) clinics is feasible and acceptable. It is unknown whether a risk‐guided model would facilitate appropriate PrEP use among MCH attendees better than universally offering PrEP.

**Methods:**

The PrEP Implementation for Mothers in Antenatal Care (PrIMA) study was a cluster randomized trial to assess two models for PrEP delivery among pregnant women seeking routine MCH care at 20 public clinics in Kenya between January 2018 and July 2019 (NCT03070600). In the Universal arm, all participants received PrEP counselling and self‐selected whether to initiate PrEP. In the Targeted arm, participants underwent an HIV risk assessment, including an objective risk‐scoring tool and an offer of HIV self‐tests for at‐home partner testing; those determined to be at high risk received a PrEP offer. Participants were followed through 9 months postpartum. Primary outcomes included incident HIV and appropriate PrEP use (defined as PrEP uptake among those at high risk and no PrEP uptake for those not at risk). Outcomes were compared using intention‐to‐treat analyses, adjusting for baseline HIV risk and marital status.

**Results:**

Among 4447 women enrolled, the median age was 24.0 years (interquartile range [IQR]: 20.9, 28.3), and most were married (84.8%). The median gestational age at enrolment was 24 weeks (IQR: 20, 30). Women in the Targeted arm were more likely to be at high risk for HIV acquisition at baseline (51.6% vs. 33.3%). During 4638 person‐years (p‐yr) of follow‐up, there were 16 maternal HIV infections with no difference in maternal HIV incidence between arms: 0.31/100 p‐yr (95% CI: 0.15, 0.65) Targeted and 0.38/100p‐yr (95% CI: 0.20, 0.73) Universal (adjusted relative risk [aRR]: 0.85 [CI: 0.28, 2.55]). There was no significant difference in the frequency of appropriate PrEP use between the arms (68.2% vs. 59.1% in Targeted vs. Universal, respectively) (aRR: 1.03 [CI: 0.96, 1.10]).

**Conclusions:**

Given comparable maternal HIV incidence and PrEP uptake in Universal and Targeted approaches, and the simplicity that universal PrEP offers, our findings suggest that universal PrEP counselling is optimal for integrating PrEP in MCH systems.

## 1 INTRODUCTION

During the pregnancy and breastfeeding periods, there is an increased risk of HIV acquisition [1, 2, 3]. Women who acquire HIV during these periods are at higher risk for vertical transmission than those with chronic HIV infection [4, 5, 6, 7]. Pre‐exposure prophylaxis (PrEP) is an effective, female‐controlled option for HIV prevention recommended for use in pregnant and breastfeeding populations [8, 9, 10, 11, 12, 13, 14, 15].

While studies have demonstrated the feasibility and acceptability of PrEP in maternal and child health (MCH) clinics, implementation questions remain [16, 17, 18, 19, 20, 21]. Introducing PrEP in MCH clinics presents challenges to overburdened healthcare workers (HCWs) [22, 23] and requires adaptations in HIV testing, PrEP counselling, dispensing and adherence counselling. Importantly, women need to understand their risk of acquiring HIV to make informed decisions about PrEP. Minimizing unnecessary PrEP use among women at low risk avoids side effects or foetal exposure and conserves PrEP resources for those at high risk. Implementation models that maximize PrEP use among women most at risk for HIV while minimizing use among low‐risk women have not been studied.

The World Health Organization recommends a risk‐guided approach for HIV prevention as part of prevention‐of mother‐to‐child transmission (PMTCT) programmes [11]. Identified risk factors for HIV acquisition in pregnancy [24] could be used to screen women based on HIV risk. Risk‐guided approaches may increase service efficiency by limiting who receives PrEP counselling and offer [25]; moreover, risk‐guided counselling could improve risk perception and PrEP acceptance and continuation [26]. Conversely, risk‐guided approaches could increase stigma or bottlenecks due to increased counselling time among “at‐risk” women. A universal model of counselling that frames reasons to consider PrEP (such as partner HIV status) to *all* pregnant women after which women decide whether to initiate PrEP may decrease stigma. However, universal PrEP offers may lead to either over‐ or under‐use of PrEP depending on how clearly women understand their risk following general counselling.

It is unclear whether a risk‐guided model would reduce HIV incidence and increase appropriate PrEP decisions than universally offering PrEP. To address this question, we conducted a cluster‐randomized trial (cRCT), the PrEP Implementation for Mothers in Antenatal Care (PrIMA) study, to compare Universal and Targeted (risk‐guided) approaches to PrEP delivery. We hypothesized that Targeted PrEP delivery would result in lower HIV incidence and more appropriate PrEP decisions.

## 2 METHODS

### 2.1 Study design

The PrIMA study was a cRCT evaluating two models of PrEP delivery among pregnant women attending MCH clinics (Clinical Trials.gov #NCT03070600). The study protocol has been described [27]. Briefly, 20 public MCH clinics in Siaya or Homa Bay counties in Kenya were randomized to Universal or Targeted PrEP counselling using restricted randomization, stratified by antenatal care clinic (ANC) volume and county. Enrolment occurred between 15 January 2018 and 31 July 2019, and all women had the chance to be followed through 9 months postpartum.

MCH clinics were eligible for inclusion if they had >350 HIV‐negative annual ANC clients and offered postnatal care. Study facilities were selected in collaboration with the County Ministry of Health (MoH) to ensure geographic distribution. Assuming a coefficient of variation (*k*) of 0.2, with 10 clinics per cluster and 200 women enrolled per facility, the study had 80% power to detect a two‐fold difference in annual HIV incidence.

#### 2.1.1 Universal arm

At Universal arm clinics, after informed consent, participants received PrEP counselling using a standardized script listing risk factors for HIV and considerations for PrEP use, after which participants decided whether to initiate PrEP.

#### 2.1.2 Targeted arm

At Targeted arm clinics, participants underwent an HIV risk assessment, adapted from Kenyan National AIDS and STI Control Programme (NASCOP) guidelines and an HIV risk assessment tool shown to predict HIV risk in pregnant and postpartum women (Pintye tool) [24]. In the Targeted arm, those at high risk received standardized PrEP counselling and offer. Although PrEP offer in the Targeted arm was risk‐guided, any woman who asked for PrEP was provided with PrEP regardless of risk status. The Pintye HIV risk assessment tool had an area under the curve of 0.76 to predict HIV incidence in pregnancy/postpartum [24]; score: 1 point per lifetime sexual partner, 6 points if the sexual partner's HIV status is positive or unknown and 5 points for syphilis positive [24]. Under NASCOP guidelines, anyone with any of the following in the last 6 months was eligible for PrEP: transactional sex, sexually transmitted infection (STI) diagnosed or treated, forced sex, physically assaulted, including by their sexual partner, shared needles during intravenous drug use or used post‐exposure prophylaxis more than twice [10]. Participants with a score of ≥6 on the assessment tool or any NASCOP risk factor were considered high risk. Targeted arm participants were offered HIV self‐test kits (HIVST) for use with partners to provide information on partner HIV status to guide PrEP decision‐making. Women were counselled that they should not accept and/or offer HIVST to their partners if they had any concerns about physical, emotional or other types of repercussions from offering HIVST to their partners. Women were asked at subsequent study visits about the partner HIVST results and about whether they had changed their decision about whether to use, or not use, PrEP.

### 2.2 Eligibility criteria

Eligibility criteria included: pregnant at enrolment, HIV negative (documented from MCH records during the ANC visit), not currently using PrEP, ≥ 15 years old, tuberculosis negative, planned to reside in the region for ≥1‐year postpartum, planned to receive postnatal care at the study facility and not enrolled in any other studies. In both arms, those with creatinine clearance (CrCl) ≤50 ml/minute or hepatitis B surface antigen (HBsAg) positivity were ineligible for PrEP per NASCOP guidelines [10].

### 2.3 Enrolment and follow‐up

At enrolment, questionnaires were administered on socio‐demographic characteristics, mental health (PHQ‐2) [28], intimate partner violence (IPV) using the HITS scale [29], PrEP knowledge and partner HIV status. In the Universal arm, participants underwent HIV risk assessment after they received PrEP counselling and had decided whether to accept PrEP. Gestational age at enrolment was assessed using last menstrual period (97.9%) or fundal height (2.1%).

Syphilis testing was conducted through rapid plasma reagin (RPR) during the first ANC visit and results were abstracted from medical records. If no syphilis data were available, the study team provided testing using SD Bioline HIV/Syphilis lateral flow Duo Test Kits.

Participants were seen at monthly ANC visits, followed by postnatal visits at 6 weeks, 14 weeks, 6 months and 9 months postpartum. Visits aligned with Kenya MoH recommendations for antenatal and infant immunization visits. Self‐reported PrEP adherence was assessed. At each follow‐up visit, participants had blood collected for HIV testing and those on PrEP had dried blood spots (DBS) collected for assessment of drug levels.

Between June and October 2018, the Kenya MoH introduced the offer of partner HIVST to women attending MCH. Information on HIVST including: the offer of HIVST to partner, partner uptake and results were collected. All data were collected using password‐protected tablets and uploaded daily to encrypted Research Electronic Data Capture (REDCap) servers [30].

### 2.4 Laboratory methods

Participants received HIV testing at all study visits following the NASCOP HIV testing algorithm using two rapid HIV‐1 antibody tests, Determine Third Generation and First Response Third Generation [31, 32]. Participants with positive or inconclusive HIV test results had confirmatory HIV‐1 DNA PCR testing, Roche Abbott and Cobas TaqMan molecular platforms the gold standard for confirmatory testing with serological enzyme‐linked immunosorbent assay fourth generation at the KEMRI‐CDC lab in Kisumu, Kenya. The study Leadership Team reviewed each positive or inconclusive HIV result to adjudicate HIV status, blinded to a randomized group. Participants who seroconverted during the study period were offered, encouraged and supported to complete infant HIV testing following NASCOP guidelines.

### 2.5 Analysis

#### 2.5.1 Primary outcomes

Maternal HIV incidence and “appropriate PrEP use” were the primary outcomes. HIV incidence was defined as confirmed maternal HIV infections per 100 person‐years of follow‐up. Incident HIV infections were compared between arms using generalized estimating equations (GEE) with a Poisson link. Appropriate PrEP use was defined as the proportion of high‐risk (either NASCOP or Pintye tools) accepting PrEP and low‐risk declining PrEP and was compared between arms using GEE with a Poisson link. Independent correlation structures with robust standard errors were used for all GEE models. All analyses included clustering by the facility. Adjusted models included baseline high‐risk HIV status and marital status.

#### 2.5.2 Secondary outcomes

PrEP acceptance, initiation, adherence, and duration, and partner HIV status (at 9 months postpartum) were secondary outcomes. PrEP acceptance was defined as participants who accepted PrEP at any visit. PrEP confirmation visit was defined as participant‐report of swallowing PrEP pills at visits after PrEP acceptance, with the PrEP initiation date defined as the median date between the PrEP acceptance and PrEP confirmation visits. At the PrEP confirmation visit, self‐reported PrEP adherence was dichotomized as no missed doses versus any missed doses in the past 30 days. The PrEP duration was defined as the time between the PrEP initiation date and discontinuation or study end [33].

The proportion of women who accepted and used PrEP and self‐reported adherence was compared between arms using GEE with a Poisson link. Proportions of partner HIV status unknown and change in partner HIV status knowledge by the study end were compared between arms using GEE with a Poisson link. Mean PrEP duration was compared between arms using GEE with a Gaussian link.

All analyses were conducted using Stata SE 17.0 (StataCorp, College Station, TX).

### 2.6 External oversight

An External Advisory Panel (EAP) met at study initiation and mid‐way through follow‐up to evaluate safety and endpoints. At the mid‐way evaluation, baseline differences were noted between trial arms. The EAP advised that analyses be adjusted for key differences at baseline (marital status and HIV risk). Two Community Advisory Boards, one in Homa Bay and one in Siaya, met at least annually to review study progress and to make recommendations.

### 2.7 Human subjects

Protocols were approved by the Kenyatta National Hospital‐University of Nairobi Ethics and Research Committee (P73/02/2017) and the University of Washington Institutional Review Board (STUDY00000438) prior to the initiation of the study. The study obtained approval from the National Commission for Science, Technology, and Innovation (NASCOTI) as well as the Kenya Pharmacy and Poisons Board. Approval to conduct study activities was obtained from the Homa Bay and Siaya County MoH and from all 20 facilities. All participants provided written informed consent. Participants under 18 years old enrolled without parental consent as pregnant people are considered emancipated minors and able to consent under Kenyan law.

## 3 RESULTS

The study team assessed 45 facilities for inclusion, 19 of which did not meet the inclusion criteria, and six of which were excluded based on recommendations of County MoH.

### 3.1 Baseline characteristics and comparison by randomization arm

**Figure 1:**
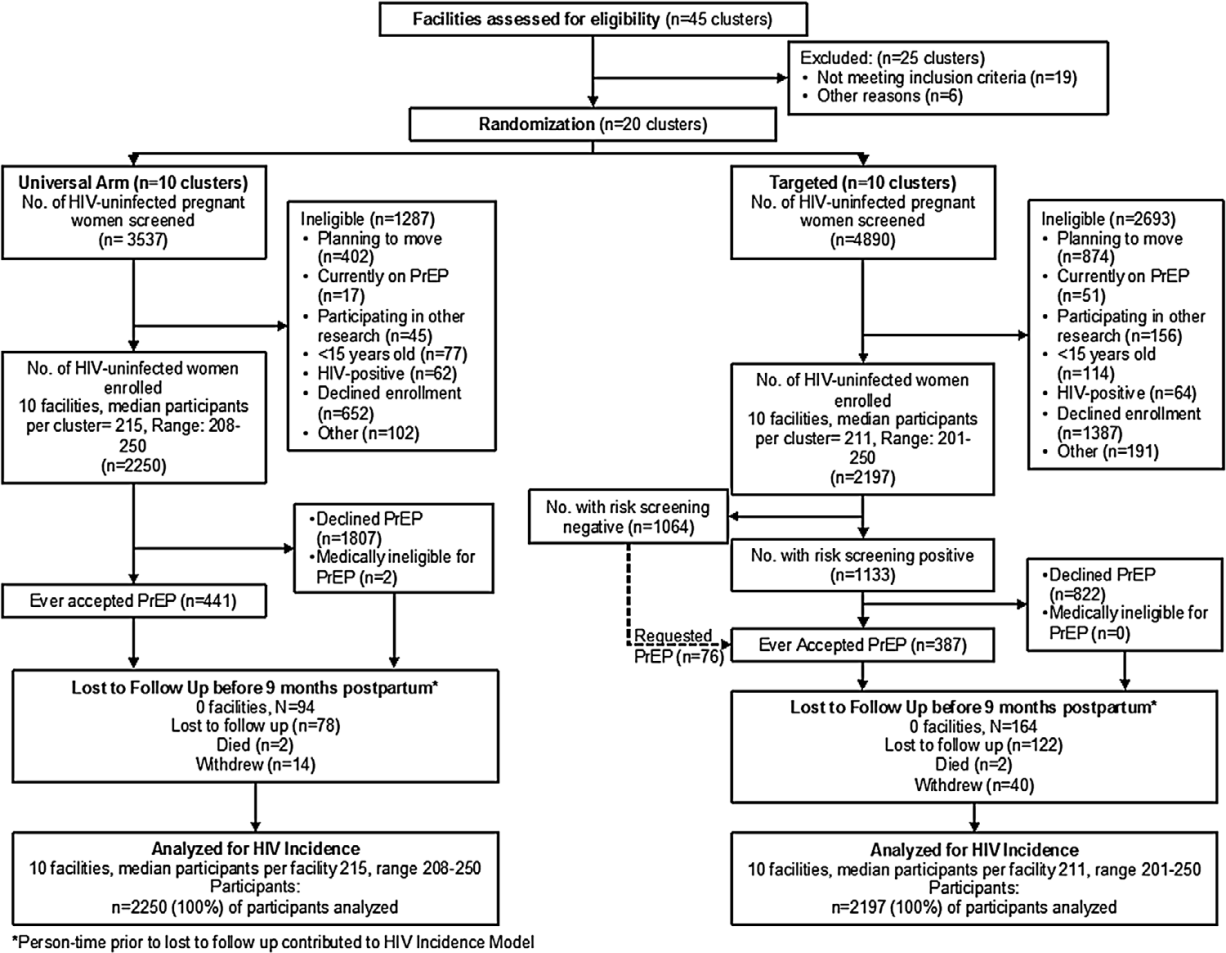
CONSORT diagram of PrIMA study.

Of 8427 women screened for enrolment, 4447 women were enrolled (2250 Universal arm and 2197 Targeted arm) (Figure 1 and Table S1). The median age of participants was 24.0 years (interquartile range [IQR] 20.9, 28.3). More than half of the participants were < 25 years old (57.3%), and most (84.8%) were currently married. Median educational attainment was 10 years (IQR 8, 12) and 14.9% of women were employed. Just under half (47.7%) reported living in crowded environments. The median gestational age at enrolment was 24 weeks (IQR 20, 30) and the majority (74.4%) of participants had been pregnant previously. Overall, 9.5% of women had symptoms of moderate‐to‐severe depression (PHQ2 ≥3) and 7.8% reported IPV (HITS score ≥10). Overall, 42.3% of participants met high‐risk criteria (Table 1). Women in the Targeted arm clinics were more likely to be married, know someone on PrEP, have depressive symptoms and be at high risk for HIV acquisition (51.6% vs. 33.3% Universal arm) than women in the Universal arm.

**Table 1:** Baseline characteristics of PrIMA study participants

		*n* (%) or Median (IQR)				
		Overall	Universal		Targeted	
	*N*	(*N* = 4447)	*N*	(*n* = 2250)	*N*	(*n* = 2197)
Demographic characteristics						
Age (years)	4445	24.0 (20.9–28.3)	2249	23.9 (20.7–28.2)	2196	24.1 (21.0–28.5)
Age category (years)	4445		2249		2196	
<25		2545 (57.3)		1307 (58.1)		1238 (56.4)
25–34		1680 (37.8)		830 (36.9)		850 (38.7)
≥35		220 (4.9)		112 (5.0)		108 (4.9)
Currently married	4394	3727 (84.8)	2231	1827 (81.9)	2163	1900 (87.8)
Education (years)	4344	10 (8, 12)	2231	10 (8, 12)	2113	10 (8, 12)
Regular employment	4372	651 (14.9)	2220	337 (15.2)	2152	314 (14.6)
People per room	4397	1.7 (1–2.5)	2233	1.8 (1.2, –2.5)	2164	1.7 (1–2.5)
2+ people per room (crowded environments)	4397	2099 (47.7)	2233	1102 (49.4)	2164	997 (46.1)
Pregnancy history						
Gestational age at enrolment (weeks)	4442	24 (20, 30)	2249	25 (20, 30)	2193	24 (19, 28)
Gestational age determined by:	4442		2249		2193	
Last menstrual period (LMP)		4347 (97.9)		2182 (97.0)		2165 (98.7)
Fundal height		95 (2.1)		67 (3.0)		28 (1.3)
Previous pregnancy	4425	3294 (74.4)	2239	1661 (74.2)	2186	1633 (74.7)
Previous abortion/miscarriage	4415	478 (10.8)	2234	217 (9.7)	2181	261 (12.0)
Prior preterm (<37 weeks)	4447	45 (1.0)	2250	15 (0.7)	2197	30 (1.4)
Risk assessment chacteristics						
No. of lifetime sexual partners	4431	2 (2, 3)	2236	2 (2, 3)	2195	3 (2, 3)
HIV status of primary sexual partner(s)	4437		2241		2196	
Positive		186 (4.2)		90 (4.0)		96 (4.4)
Negative		2816 (63.5)		1615 (72.1)		1201 (54.7)
Unknown		1380 (31.1)		497 (22.2)		883 (40.2)
No male partner		55 (1.2)		39 (1.7)		16 (0.7)
Partner HIV status unknown among participants with a partner	4382	1380 (31.5)	2202	497 (22.6)	2180	883 (40.5)
Partner on ART if HIV positive	178	172 (96.6)	87	85 (97.7)	92	87 (95.6)
Syphilis positive	4365	45 (1.0)	2196	18 (0.8)	2169	27 (1.2)
In the last 6 months:						
Sex for money/favours	4426	78 (1.8)	2239	37 (1.7)	2187	41 (1.9)
Diagnosed or treated for STI	4426	113 (2.6)	2240	37 (1.7)	2186	76 (3.5)
Forced to have sex	4428	249 (5.6)	2239	118 (5.3)	2189	131 (6.0)
Physically assaulted	4429	264 (6.0)	2241	147 (6.6)	2188	117 (5.3)
Shared needles during IDU	4428	9 (0.2)	2241	1 (0.0)	2187	8 (0.4)
Used PEP >2 times	4426	20 (0.5)	2240	5 (0.2)	2186	15 (0.7)
High risk by NASCOP assessment	4447	563 (12.7)	2250	277 (12.3)	2197	286 (13.0)
High risk by Pintye assessment	4447	1639 (36.9)	2250	617 (27.4)	2197	1022 (46.5)
High risk score by either assessment	4447	1883 (42.3)	2250	750 (33.3)	2197	1133 (51.6)
Psychosocial factors						
Heard of PrEP before	4409	2188 (49.6)	2232	1184 (53.0)	2177	1004 (46.1)
PHQ‐2 score ≥ 3	4141	393 (9.5)	2113	109 (5.2)	2208	284 (14.0)
HITS score ≥ 10	4422	345 (7.8)	2237	147 (6.6)	2185	198 (9.1)

Abbreviations: ART, antiretroviral therapy; IDU, injection drug use; PEP, post‐exposure prophylaxis; PHQ‐2, Patient Health Questionnaire‐2; STI, sexually transmitted infection.

### 3.2 Primary outcomes

During 4638 person‐years of follow‐up, there were 16 maternal HIV infections; an overall HIV incidence of 0.35 infections/100 person‐yrs (p‐yrs) (95% confidence interval [CI]: 0.21, 0.56). HIV incidence in the two arms was similar: seven incident maternal HIV infections (0.31/100 p‐yrs [CI: 0.15, 0.65]) in the Targeted arm and nine incident infections (0.38/100 p‐yrs [CI: 0.20, 0.73]) in the Universal arm (Table 2). The adjusted relative risk (aRR) for HIV incidence in the Targeted versus Universal arm was 0.85 (CI: 0.28, 2.55, *p*: 0.77).

**Table 2:** PrEP uptake, adherence and HIV incidence by study arm

		*n* (%) or Median (IQR)					
	*N*	Overall (*N* = 4447)	Universal (*n* = 2250)	Targeted (*n* = 2197)	RR (95% CI) *p*‐value		aRR^a^ (95% CI) *p*‐value
Primary outcomes							
HIV incidence (PCR confirmed)							
Person‐time (years)	4447	4637.6	2371.6	2266.0	0.81 (0.29–2.31) *p* = 0.70		0.85 (0.28–2.55) *p* = 0.77
HIV events	4447	16	9	7			
Incidence/100py (95% CI)	4447	0.35 (0.21–0.56)	0.38 (0.20–0.73)	0.31 (0.15–0.65)
Appropriate PrEP decision							
Appropriate PrEP decision	4447	2834 (63.7)	1535 (68.2)	1299 (59.1)	0.87 (0.69–1.09) *p* = 0.23		1.03 (0.96–1.10) *p* = 0.37
High risk, declined PrEP		1334 (30.0)	512 (22.8)	822 (37.4)			
High risk, accepted PrEP		549 (12.3)	238 (10.6)	311 (14.2)			
Low risk, accepted PrEP		279 (6.3)	203 (9.0)	76 (3.5)			
Low risk, declined PrEP		2285 (51.4)	1297 (57.6)	988 (45.0)			
Among high risk, accepted PrEP	1883	549 (29.2)	238 (31.7)	311 (27.4)	0.86 (0.61–1.23) *p* = 0.42		0.87 (0.61–1.24) *p* = 0.43
Among low risk, accepted PrEP	2564	279 (10.9)	203 (13.5)	76 (7.1)	0.53 (0.24–1.18) *p* = 0.12		0.52 (0.24–1.13) *p* = 0.10
Among low risk, declined/did not request PrEP	2564	2285 (89.1)	1297 (86.5)	988 (92.9)	1.07 (0.99–1.17) *p* = 0.10		1.08 (0.99–1.17) *p* = 0.084
Secondary outcomes							
PrEP uptake							
Accepted PrEP	4447	828 (18.6)	441 (19.6)	387 (17.6)	0.90 (0.61–1.33) *p* = 0.59		0.74 (0.50–1.10) *p* = 0.14
Initiated PrEP	4447	720 (16.2)	397 (17.6)	323 (14.7)	0.83 (0.57–1.22) *p* = 0.35		0.68 (0.46–1.02) 0.062
High risk, initiated PrEP	1883	471 (25.0)	213 (28.4)	258 (22.8)	0.80 (0.57–1.13) *p* = 0.21		0.80 (0.56–1.13) *p* = 0.21
Low risk, initiated PrEP	2564	249 (9.7)	184 (12.3)	65 (6.1)	0.50 (0.22–1.15) *p* = 0.10		0.48 (0.21–1.10) *p* = 0.082
PrEP persistence and adherence (among women who initiated PrEP)							
PrEP duration (months)^b^	720	8.9 (3.5–11.6)	8.6 (3.2–11.4)	9.0 (3.8–11.9)	0.18^c^ (−0.83 to 1.20) *p* = 0.71		0.11^c^ (−0.94 to 1.15) *p* = 0.83
Ever discontinued PrEP use	720	365 (50.7)	208 (52.4)	157 (48.6)	0.93 (0.72–1.20) *p* = 0.57		0.93 (0.71–1.22) *p* = 0.60
Never		355 (49.3)	189 (47.6)	166 (51.4)			
Discontinued and stayed off		304 (42.2)	171 (43.1)	133 (41.2)			
Discontinued and restarted		61 (8.5)	37 (9.3)	24 (7.4)			
Self‐reported perfect PrEP adherence in the past 30 days at PrEP confirmation visit	710	406 (57.2)	206 (52.8)	200 (62.5)	1.18 (0.96–1.46) *p* = 0.12		1.13 (0.91–1.41) *p* = 0.28

^a^ Adjustment factors include marital status and baseline HIV risk status.

^b^ PrEP duration to the first reported discontinuation of PrEP.

^c^ Risk difference and adjusted risk difference.

Overall, 2834 (63.7%) had an appropriate PrEP decision, with non‐significantly lower frequency in the Targeted than Universal arm (59.1% vs. 68.2%) (aRR: 1.03 [CI: 0.96, 1.10] *p*: 0.37). Among 1883 women determined to be at risk, 29.2% accepted PrEP; in the Targeted arm, 311 (27.4%) women at risk accepted PrEP which was lower than the 238 (31.7%) of the Universal arm (aRR 0.87 [CI: 0.61–1.24] *p* = 0.43). Among low‐risk women, 76 (7.1%) of 1064 participants in the Targeted arm independently requested PrEP, a lower frequency than in the Universal arm (203/1500 [13.5%]) (aRR 0.52 [CI: 0.24–1.13] *p*: 0.10). Only two participants in the study were medically ineligible for PrEP, both due to an Hepatitis B (HBV) diagnosis. None were ineligible due to creatinine clearance levels.

### 3.3 Secondary outcomes

Overall, 828 women (18.6%) accepted PrEP and there was no significant difference between arms for PrEP acceptance (aRR: 0.74 [CI: 0.50, 1.10] *p*: 0.14). Overall, 720 (16.2%) women initiated PrEP, with a trend towards statistical significance for a lower likelihood of PrEP initiation in the Targeted arm (aRR: 0.68 [CI: 0.46, 1.02] *p*: 0.062) (Table 2). Of those who initiated PrEP, the median duration of PrEP use was 9.0 months (IQR: 3.8, 11.9) in the Targeted arm and 8.6 months (IQR: 3.2, 11.4) in the Universal arm, with no difference between arms (adjusted risk difference: 0.11 [CI: –0.94, 1.15] *p*: 0.83). Approximately half of those who initiated PrEP (49.3%) continued use throughout follow‐up, with similar PrEP continuation in both arms (aRR: 0.93 [CI: 0.71, 1.22] *p*: 0.60). Just over half of the participants (52.8% Universal, 62.5% Targeted) reported perfect PrEP adherence at their first study visit after accepting PrEP (aRR: 1.13 [CI: 0.91, 1.41] *p*: 0.28).

### 3.4 HIVST uptake in Targeted arm

In the Targeted arm, 1384 (63.5%) accepted HIVST for partner HIV testing (Table 3). Women who accepted partner HIVST more often reported living with their partner (89.9% vs. 84.6%, *p*: 0.006). Women who declined partner HIVST were more likely to have a partner with known HIV‐positive status (10.3% declined vs. 1.0% accepted, *p*<0.001) and reported older partners than those who accepted HIVST (median 31 vs. 30, *p*: 0.006). Among women who accepted HIVST, most (85.8%) offered the HIVST to their partners, of whom almost all (96.0%) reported their partners used the tests and among those who reported their partners used the HIVST, 98.9% reported seeing their partner's results themselves. HIVST results identified 12 (1.1%) partners with HIV‐positive results and three (0.3%) with indeterminate results. HIVST results decreased the proportion of women with unknown partner status from 40.5% at baseline to 21.7% by 9 months postpartum (*p* = 0.002) in the Targeted arm compared to the Universal arm where unknown partner status did not change (22.6–23.5%; *p* = 0.76) (Table 4).

**Table 3:** Participant characteristics by HIV self‐test acceptance and self‐test use in Targeted arm among women with male partners

	*n* (%) or Median (IQR)				
		Self‐test at enrolment			*p*‐value^a^
	*N*	Overall (*n* = 2179)	Accepted (*n* = 1384)	Declined (*n* = 795)	
Enrolment characteristics					
Partner HIV status^b^					
Positive	2179	96 (4.4)	14 (1.0)	82 (10.3)	<0.001
Negative	2179	1200 (55.1)	770 (55.6)	430 (54.1)	0.88
Unknown	2179	883 (40.5)	600 (43.4)	283 (35.6)	0.43
HIV tested as a couple in the past	1982	1167 (58.9)	737 (57.5)	430 (61.3)	0.64
Partner age	1777	30 (26–35)	30 (26–35)	31 (26–37)	0.006
Partner 10 or more years older than participant	1776	256 (14.4)	156 (13.1)	100 (17.1)	0.063
Living together with partner	1983	1745 (88.0)	1147 (89.9)	598 (84.6)	0.006
Married to partner	1988	1827 (91.9)	1188 (92.7)	639 (90.4)	0.15
Initiated PrEP	2179	320 (14.7)	201 (14.5)	119 (15.0)	0.91
PrEP initiator with partner HIV status unknown	883	157 (17.8)	114 (19.0)	43 (15.2)	0.18
HIV self‐test use and results					
Self‐tests ever distributed by participants to sexual partners	1286		1104 (85.8)		
Sexual partners who ever used a self‐test	1097		1053 (96.0)		
Partner and participant took self‐test together at visit of first used	1049		1029 (98.1)		
Did the participant see the results of the self‐test at visit of first used?^a^	1051				
No, participant doesn't know			1 (0.1)		
Yes, participant observed it			1039 (98.9)		
Yes, partner shared it			11 (1.0)		
Partner self‐test results at visit of first used	1053				
Positive			12 (1.1)		
Negative			1036 (98.4)		
Indeterminate			3 (0.3)		
Refused to answer			2 (0.2)		
Initiated PrEP following HIVST test positive or indeterminate results	15		5 (33.3)		

Abbreviation: HIVST, HIV self‐test.

^a^ Among participants whose partners used HIVST.

^b^ Three separate models were run for partner HIV status (positive vs. not positive, negative vs. not negative and unknown vs. not unknown).

**Table 4:** Participant risk and partner status knowledge at 9 months postpartum

		*n* (%) or Median (IQR)				
	*N*	Overall (*N* = 4447)	Universal (*n* = 2250)	Targeted (*n* = 2197)	RR (95% CI) *p*‐value	aRR^a^ (95% CI) *p*‐value
Partner HIV status at 9 months postpartum						
HIV status of primary sexual partner(s) at 9‐month visit	4185					
Positive		156 (3.7)	73 (3.4)	83 (4.1)	–	–
Negative		2768 (66.1)	1476 (68.5)	1292 (63.6)	–	–
Unknown		857 (20.5)	475 (22.0)	382 (18.8)	–	–
No male partner		404 (9.7)	131 (6.1)	273 (13.4)	–	–
Unknown partner HIV status at 9‐month visit^b^	3781	857 (22.7)	475 (23.5)	382 (21.7)	0.93 (0.42–2.03) *p* = 0.85	0.78 (0.40–1.51) *p* = 0.46
Partner on ART if HIV positive	72	68 (94.4)	37 (94.9)	31 (93.9)	0.99 (0.87–1.13) *p* = 0.88	0.97 (0.86–1.08) *p* = 0.57
Partner HIV status changed from unknown at baseline to known at 9‐month visit^c^	1141	676 (59.2)	220 (51.2)	456 (64.1)	1.25 (0.76–2.06) *p* = 0.37	1.25 (0.76–2.06) *p* = 0.37
Risk status at 9 months postpartum						
High risk score by either assessment at 9 months postpartum	4188	1291 (30.8)	654 (30.3)	637 (31.4)	1.03 (0.59–1.81) *p* = 0.91	0.85 (0.55–1.31) *p* = 0.46
Appropriate PrEP decision	4188	2902 (69.3)	1471 (68.2)	1431 (70.4)	1.03 (0.85–1.25) *p* = 0.76	1.09 (0.93–1.29) *p* = 0.28

Abbreviation: ART, antiretroviral therapy.

^a^ Adjustment factors include marital status and baseline HIV risk status.

^b^ Among participants with a partner at 9 months postpartum.

^c^ Among participants with an unknown partner HIV status and high risk score at enrolment.

Among 157 women who initiated PrEP and reported a partner of unknown HIV status at baseline, 114 (72.6%) accepted HIVST for partner testing and 79 (69.3%) subsequently reported their partner was HIV negative. Of these 79 women (PrEP initiators with previously unknown partner HIV status but now known HIV‐negative partner), 49 (62.0%) discontinued PrEP following partner HIVST results. Of the 15 women whose partner was newly determined to be HIV positive or indeterminate, five (33%) had initiated PrEP prior to HIVST results and five (33%) elected to start PrEP after HIVST results, and five (33%) did not start PrEP. Because the NASCOP implemented HIVST in MCH over the course of the study, we ascertained HIVST acceptance in both study arms. However, few women in the Universal arm (0.93%, [21/2250]) reported accepting HIVST through MCH due to stockouts, lack of systematic offer and low awareness.

### 3.5 Participant risk characteristics at 9 months postpartum

Using new information available at the end of the study to estimate risk status, 30.8% of women overall would be classified as high risk, 637/2032 (31.3%) in the Targeted arm and 654/2156 (30.3%) in the Universal arm (Table 4). Thus, the proportion of “high risk” women decreased from baseline (51.6%) to 9 months (31.3%) (*p* = 0.001) in the Targeted arm and from 33.3% to 30.3% in the Universal arm (*p* = 0.26). With the significant decrease in the proportion of women defined as being in the “high risk” category at 9 months postpartum within the Targeted arm, there was no longer an increased relative risk of a participant being in the “high risk” category at 9 months postpartum between the Targeted and Universal arms (aRR: 0.85 [CI: 0.55–1.31] *p*: 0.46). Based on 9‐month postpartum partner HIV status knowledge, the proportion who made appropriate PrEP decisions was 69.3% (2902/4188) overall, 1431/2032 (70.4%) in the Targeted arm and 1471/2156 (68.2%) in the Universal arm (aRR: 1.09 [CI: 0.93–1.290 *p* = 0.28).

## 4 DISCUSSION

In this cRCT, we found that a risk‐guided PrEP offer (Targeted) was not superior to a Universal PrEP offer, based on appropriate PrEP decision and HIV incidence, among women attending MCH in high HIV prevalence regions of Kenya. The frequency of appropriate PrEP decisions did not differ significantly between the study arms, HIV incidence was low in both arms and PrEP continuation was high. These results demonstrate that PrEP decision‐making was not improved by risk‐guided counselling. Universal PrEP offer following simple standard counselling resulted in a comparable proportion of women making appropriate PrEP decisions as the risk‐guided approach.

Maternal HIV incidence in this cohort was approximately seven‐fold lower than in a prior study, of pregnant and postpartum women in western Kenya conducted between 2011 and 2013 (maternal HIV incidence of 2.31/100 py) [34]. Lower maternal HIV incidence likely reflects wider population antiretroviral therapy use and PrEP use in this maternal cohort. The low maternal HIV incidence that we observed is encouraging and provides the first‐of‐its‐kind data on HIV incidence in MCH clinics providing PrEP. Without PrEP, maternal HIV incidence remains a leading contributor to new paediatric HIV infections in high HIV prevalence settings [4, 5]. Our data support efforts to integrate PrEP into PMTCT programmes.

We found that almost two‐thirds of women made appropriate decisions regarding PrEP use in either study arm. In a qualitative study of HCW perspectives on delivering PrEP to pregnant and postpartum women, it was noted that risk‐guided counselling took time and uncovered issues that were sometimes challenging or uncomfortable to discuss [35]. Women may prefer not to discuss risk factors with health workers because such discussions may be stigmatizing or uncomfortable [36, 37]. Comparable PrEP uptake in the Universal arm suggests that the general counselling was sufficient for women to privately deliberate their PrEP decision. Among low‐risk women, a slightly higher proportion of PrEP uptake in the Universal arm (12.3% vs. 6.1%, *p*: 0.082) may reflect undisclosed risk factors, imprecision in the assessment of risk or unclear messaging about PrEP that could be addressed in counselling approaches.

Our results suggest a Universal approach may be the simplest and most effective option for PrEP counselling in MCH as no screening is required to reach similar levels of appropriate PrEP use and HIV incidence as a risk‐guided strategy. A Universal PrEP offer approach lends itself to group counselling strategies and may allow for task shifting to reduce the burden on nurses. Universal PrEP offer allows women to control all aspects of the PrEP cascade, including assessing their own risk and deciding whether PrEP is the right option. Universal PrEP offer may reduce stigma as it is being generally discussed and not targeted to certain women. Universal PrEP counselling also increases community awareness of PrEP, which can influence PrEP decision‐making and reduce stigma [38, 39]. Our results also demonstrate low rates of ineligibility due to CrCl ≤50 ml/minute or HBSAg positivity suggesting that universal screening for CrCl or HBSAg is unnecessary [40].

Our Targeted arm strategy included the provision of HIVST for partner testing. We found high uptake (>60%) of HIVST with very high levels of partner use (>80%). The greatest drop off in HIVST use was at the point of HIVST acceptance by participants, when participants were advised not to accept or offer HIVST to their partners if there were concerns about IPV or negative repercussions. Based on this, additional research is needed to identify strategies to support participants at risk of IPV in partner HIV testing. Almost 100% of women whose partner tested reported seeing the results with their partner. In the Targeted arm, the proportion of women who did not know their partner's status declined from >40% to 18.8% by the end of the study, the majority of cases identified an HIV‐negative partner and decreased the likelihood of being classified “high risk.” At enrolment, some women in the Targeted arm classified as high risk due to unknown partner status correctly speculated that their partner was HIV negative and declined PrEP. HIVST helped women who initiated PrEP discontinue PrEP if they found their partner was negative. Others with a newly identified HIV‐negative partner may have continued PrEP due to having other partners or suspicions of infidelity. The HIVST uptake and impact in the Targeted arm suggests that offering HIVST in MCH clinics for partner testing along with a Universal PrEP offer could be an important strategy.

Future implementation science research will be useful to refine strategies for Universal PrEP counselling in MCH including determining whether task shifting of components of the PrEP delivery cascade is appropriate [21]. Similarly, research will be needed to understand approaches to support the implementation of new long‐acting PrEP formulations during pregnancy and breastfeeding once these regimens are approved for use in this context.

This study has strengths and limitations. This was a large multisite cRCT with high retention (94%). We included HIV PCR confirmatory testing for all potential new maternal HIV infections [32]. Low HIV incidence in both arms limited power for comparisons of that primary outcome; however, the similarity in HIV incidence in both arms (0.38 and 0.31/100 person‐years) suggests that meaningful differences are unlikely. Changing MoH policies and the COVID‐19 pandemic also affected study operations. MoH‐led HIVST rollout could have influenced participant knowledge of their partners’ HIV status and PrEP acceptance overall; however, only 0.93% of women in the Universal arm reported receiving HIVST, demonstrating limited programmatic rollout. Participant retention, outcome ascertainment and health‐seeking behaviour were minimally impacted by COVID‐19‐related restrictions. Finally, our observed rates of PrEP adherence and persistence could have been influenced by efforts to optimize study retention, and our estimates of PrEP adherence rely on self‐report, which can overestimate adherence.

## 5 CONCLUSIONS

The results of the PrIMA study suggest that incorporating HIV‐risk screening to target PrEP offer to high‐risk women in ANC does not influence HIV incidence or appropriate PrEP uptake compared with universal PrEP counselling to all women receiving antenatal care.

## COMPETING INTERESTS

JMB is employed by Gilead Sciences, outside of this work. BAR serves on Data Safety Monitoring Boards for Gilead Science, outside of this work. All other authors declare no competing interests.

## AUTHORS’ CONTRIBUTIONS

GJS, JMB, JK, ADW and JP conceived and designed the study. JCD, NN, BO, LG, FA, SW, MM and DO collected the data. JS, JCD, JP and BAR analysed the data. JCD and LG verified the data and analysis. JCD and GJS drafted the manuscript. All authors critically reviewed the manuscript.

## FUNDING

Funding for this project was provided by the National Institutes of Health, National Institute of Allergy and Infectious Disease (GJS‐R01AI125498) and Eunice Kennedy Shriver National Institute of Child Health & Human Development (GJS‐R01HD094630; JP‐R01HD100201). The team was supported by the University of Washington's Center for AIDS Research Behavioral Sciences Core and Biometrics Core (P30AI027757) and the Global Center for the Integrated Health of Women, Adolescents, and Children (Global WACh). The funders had no role in study design, data collection and analysis, decision to publish or preparation of the manuscript.

## Supporting information


**Table S1**: Distribution of demographic characteristics of women screened and enrolled in the study

## Data Availability

Data are available indefinitely at https://github.com/jcdettin/PrIMA‐PrimaryOutcomes.git
