# Supplementary material for: Risk‐based versus universal PrEP delivery during pregnancy: a cluster randomized trial in Western Kenya from 2018 to 2019
Source: J Int AIDS Soc. 2023 Feb 20;26(2):e26061. doi: 10.1002/jia2.26061 (PMC9939942; doi:10.1002/jia2.26061)
Supplement: Supplementary file 1 — Table S1: Distribution of demographic characteristics of women screened and enrolled in the study [file JIA2-26-e26061-s001.docx]

**Supplementary Table 1. Distribution of demographic characteristics of women screened and enrolled in the study**

|  | **n (%) or Median (IQR)** | | | | | |  |
| --- | --- | --- | --- | --- | --- | --- | --- |
|  | **Project arm** | | | | | |  |
|  | **Universal** | | | **Targeted** | | | |
|  | **Screened overall (N=3537)** | **Screened Not enrolled (N=1287)** | **Enrolled (n=2250)** | **Screened overall (N=4890)** | **Screened not enrolled (N=2693)** | **Enrolled (n=2197)** | |
| **Demographic characteristics** |  |  |  |  |  |  | |
| Age (years) |  |  |  |  |  |  | |
| Median | 24 (20, 28) | 24 (20, 28) | 23 (20, 28) | 24 (21, 28) | 24 (21, 28) | 24 (21, 28) | |
| <25 | 1983 (56) | 687 (54) | 1296 (58) | 2690 (55) | 1490 (55) | 1200 (55) | |
| 25-34 | 1353 (38) | 515 (40) | 838 (37) | 1931 (40) | 1043 (39) | 888 (40) | |
| ≥35 | 194 (5) | 82 (6) | 112 (5) | 263 (5) | 155 (6) | 108 (5) | |
| Gestational age (weeks) | 26 (20, 31) | 27 (20, 32) | 26 (20, 30) | 25 (20, 30) | 26 (20, 32) | 24 (19, 28) | |
| Plan to receive postnatal care services at this facility | 3012 (90) | 774 (69) | 2238 (100) | 3749 (83) | 1559 (67) | 2190 (100) | |
| Percent who screened as eligible | 2902 (82) | 652 (51) | -- | 3584 (73) | 1387 (52) | -- | |
| Ratio of eligible to enrolled | 1·29 | -- | -- | 1·63 | -- | -- | |
